# In type 2 diabetes, randomisation to advice to follow a low-carbohydrate diet transiently improves glycaemic control compared with advice to follow a low-fat diet producing a similar weight loss

**DOI:** 10.1007/s00125-012-2567-4

**Published:** 2012-05-06

**Authors:** H. Guldbrand, B. Dizdar, B. Bunjaku, T. Lindström, M. Bachrach-Lindström, M. Fredrikson, C. J. Östgren, F. H. Nystrom

**Affiliations:** 1 Department of Medical and Health Sciences, Faculty of Health Science, Linköping University, SE 581 85 Linköping, Sweden; 2 Diabetes Research Centre, Faculty of Health Science, Linköping University, Linköping, Sweden; 3 Department of Clinical and Experimental Medicine, Faculty of Health Science, Linköping University, Linköping, Sweden

**Keywords:** Blood glucose, Dietary intervention, HDL-cholesterol, LDL-cholesterol, Low-carbohydrate diet, Type 2 diabetes

## Abstract

**Aims/hypothesis:**

The study aimed to compare the effects of a 2 year intervention with a low-fat diet (LFD) or a low-carbohydrate diet (LCD), based on four group meetings to achieve compliance.

**Methods:**

This was a prospective randomised parallel trial involving 61 adults with type 2 diabetes consecutively recruited in primary care and randomised by drawing ballots. Patients that did not speak Swedish could not be recruited. The primary outcomes in this non-blinded study were weight and HbA_1c_. Patients on the LFD aimed for 55–60 energy per cent (E%) and those on LCD for 20 E% from carbohydrate.

**Results:**

The mean BMI and HbA_1c_ of the participants were 32.7 ± 5.4 kg/m^2^ and 57.0 ± 9.2 mmol/mol, respectively. No patients were lost to follow-up. Weight loss did not differ between groups and was maximal at 6 months: LFD −3.99 ± 4.1 kg (*n* = 31); LCD −4.31 ± 3.6 kg (*n* = 30); *p* < 0.001 within groups. At 24 months, patients on the LFD had lost −2.97 ± 4.9 kg and those on LCD −2.34 ± 5.1 kg compared with baseline (*p* = 0.002 and *p* = 0.020 within groups, respectively). HbA_1c_ fell in the LCD group only (LCD at 6 months −4.8 ± 8.3 mmol/mol, *p* = 0.004, at 12 months −2.2 ± 7.7 mmol/mol, *p* = 0.12; LFD at 6 months −0.9 ± 8.8 mmol/mol, *p* = 0.56). At 6 months, HDL-cholesterol had increased with the LCD (from 1.13 ± 0.33 mmol/l to 1.25 ± 0.47 mmol/l, *p* = 0.018) while LDL-cholesterol did not differ between groups. Insulin doses were reduced in the LCD group (0 months, LCD 42 ± 65 E, LFD 39 ± 51 E; 6 months, LCD 30 ± 47 E, LFD 38 ± 48 E; *p* = 0.046 for between-group change).

**Conclusions/interpretation:**

Weight changes did not differ between the diet groups, while insulin doses were reduced significantly more with the LCD at 6 months, when compliance was good. Thus, aiming for 20% of energy intake from carbohydrates is safe with respect to cardiovascular risk compared with the traditional LFD and this approach could constitute a treatment alternative.

**Trial registration:**

ClinicalTrials.gov NCT01005498

**Funding:**

University Hospital of Linköping Research Funds, Linköping University, the County Council of Östergötland, and the Diabetes Research Centre of Linköping University

**Electronic supplementary material:**

The online version of this article (doi:10.1007/s00125-012-2567-4) contains peer-reviewed but unedited supplementary material, which is available to authorised users.

## Introduction

The prevalence of type 2 diabetes is increasing worldwide, and this is likely to be a consequence of the increasing prevalence of obesity. Weight loss in obesity generally leads to improvement in cardiovascular risk factors and glycaemic control [1, 2]. However, few randomised studies have specifically targeted type 2 diabetes to compare the effect of different diets in this respect. Traditionally, a low-fat diet (LFD) has been recommended [3] to patients with type 2 diabetes as a means to lose weight and, in particular, a low intake of saturated fat has been advocated [4, 5]. Interestingly, in a randomised 2 year study from Israel that achieved good compliance, a high-fat diet was shown to induce better weight reduction and improved blood lipid levels than a traditional LFD in obese participants, while the subgroup of patients with diabetes who were randomised to the high-fat diet exhibited the largest reduction in HbA_1c_ levels [6].

From a physiological point of view it could be argued that carbohydrate should be avoided to achieve good glycaemic control in type 2 diabetes, as a typical feature of type 2 diabetes is the combination of reduced insulin sensitivity and the failure of beta cells to provide adequate amounts of insulin to handle glucose derived from carbohydrate in the diet. However, when the macronutrient composition is changed in a diet by reducing carbohydrate, the energy from this source is primarily replaced by that from fat, as a high energy intake from protein is hard to achieve in the long term. Thus, a low-carbohydrate diet (LCD) is quite similar to one with a high intake of fat, which has traditionally been linked with increased risk for arteriosclerosis, particularly when large amounts of saturated fat are consumed. However, recent data have challenged the concept of the risks with a high-fat diet. In a Swedish observational study in 28,000 middle-aged individuals, neither a high fat intake nor an intake of large amounts of saturated fat (22 E%) was linked with an increased risk for cardiovascular disease [7, 8].

Most earlier studies examining high-fat diets in patients with type 2 diabetes have had limitations, such as a high dropout frequency [9–12], lack of randomisation [13–15], or a maximum duration of 12 months [11, 12, 16]. Another problem for the clinical feasibility of studies involving patients with type 2 diabetes has been the scale of the resources necessary to achieve compliance with the tested diets; typically, numerous individual meetings with trained dietitians are held during the studies [11, 16, 17]. If these measures were to become part of routine healthcare for such a common disease, there would be considerable economic implications for the medical provider.

We performed a randomised study in patients with type 2 diabetes to compare glycaemic control, weight loss and cardiovascular risk factors based on the advice of a low-carbohydrate or a traditional LFD. In contrast to most previous studies, the patients randomised to the LCD were not asked to avoid saturated fat. The interventions were based on four group meetings with a duration of 60 min each for the first year; no further group meetings during the remaining 12 months were held. Our hypothesis was that the high-fat diet would improve glycaemic control more efficiently than the traditional LFD in a study in which few resources were allocated for achieving compliance, so that clinical use of the protocol would be realistic for many providers of care.

## Methods

The study was conducted in two primary healthcare centres in the cities of Motala and Borensberg, located in southeast Sweden. Patients who fulfilled the criteria for participation were contacted individually by one of three study nurses. The nurses had also been responsible for the care of these potential participants ahead of the study start. The inclusion criteria were a diagnosis of type 2 diabetes treated with diet with or without additional oral glucose-lowering medication, incretin-based therapy or insulin. There were no weight or age exclusion criteria, but patients who had difficulties understanding the Swedish language, were suffering from severe mental disease or malignant disease, or who were abusing drugs could not participate in the study.

The patients were randomised to either an LCD or a traditional LFD, both with an energy content of 6,694 kJ/day (1,600 kcal/day) for women or 7,531 kJ/day (1,800 kcal/day) for men. Randomisation was not stratified and was based on drawing blinded ballots. The LCD had an energy content where 50 energy per cent (E%) was from fat, 20 E% was from carbohydrate and 30 E% was from protein. The LFD had a nutrient composition that was similar to that traditionally recommended for the treatment of type 2 diabetes in Sweden, with 30 E% from fat (less than 10 E% from saturated fat), 55–60 E% from carbohydrate and 10–15 E% from protein.

Group information was used to inform the randomised patients about which food items to choose from, and this was given at baseline, and 2, 6 and 12 months by two different physicians. One dedicated dietitian provided the participants from both groups with suitable recipes at each group meeting, and was also available consecutively during the trial for questions from the participants. However, all the information necessary was provided at the group information meetings, and thus no individual meetings with the dietitian were scheduled as part of the general protocol. Menus for 1 week were provided to the participants as meal suggestions by the dietitian. Each patient had the same dedicated nurse during the whole study period and the nurses could also provide information about food to the patients during regular consultations. The patients were recommended to check plasma glucose levels before and after meals after initiation of the study to allow for proper adjustment of medication to avoid hypoglycaemia. No information was given to change the level of physical activity. As the duration of the trial was 2 years, and because individuals with high risk for cardiovascular events were recruited, it was judged to be unethical not to be allowed to adjust medication to avoid cardiovascular disease in the study. The physician responsible for each patient at the primary healthcare centre was thus allowed to adjust hypolipidaemic and antihypertensive medications consecutively in the trial.

Investigations of anthropometrics and laboratory tests were performed at baseline and at 6, 12 and 24 months, and patients were also asked to fill out questionnaires of wellbeing at these time points. Diet records were also performed at these four visits, with one additional recording at 3 months. The diet records were conducted during 3 consecutive days, of which 1 day was a Saturday or a Sunday. The participants were provided with dedicated scales and notebooks from the organisers with which to weigh and record all food items that were consumed during these periods (food frequency questionnaires were not used). Sagittal abdominal diameter was measured with a sliding beam set square as the highest abdominal level above the upper surface of the corresponding bed. The laboratory tests were analysed at the Department of Clinical Chemistry at the University Hospital of Linköping as part of clinical routine analyses, and fasting LDL-cholesterol was thus calculated using the Friedewald formula.

### Statistics

Statistical calculations were made using PASW 18.0 software (SPSS, Chicago, IL, USA). Linear correlations were calculated as stated in the text. Comparisons within and between groups were made with Student’s paired and unpaired two-tailed *t* tests or as stated in the results section. The mean (SD) is given unless otherwise stated. Statistical significance was considered to be present at the 5% level (*p* ≤ 0.05). ANOVA with repeated measures was used for calculations of the changes during the total duration of the study.

The size of the study was based on an earlier 6 month pilot study of 28 participants with type 2 diabetes who were randomised to the same diets as in the study presented in this paper. Twenty individuals completed the pilot study and both diet groups achieved similar weight reductions, while HbA_1c_ levels tended to be lowered in the low-carbohydrate group only, without taking change in medication into account (low carbohydrate, *p* = 0.068; low-fat group, *p* = 0.8). Based on these results, the study sample was increased to at least 30 individuals in each group in the present study. None of the participants in the pilot study participated in the trial presented in this paper. The study duration of 24 months was requested by the Regional Ethics Committee of Linköping.

### Ethical approval

The study was approved by the Regional Ethics Committee of Linköping and performed in accordance with the Declaration of Helsinki. Written informed consent was obtained from all participating individuals. The study was registered with trial number NCT01005498 at ClinicalTrials.gov.

## Results

### Total study population

**Fig. 1:**
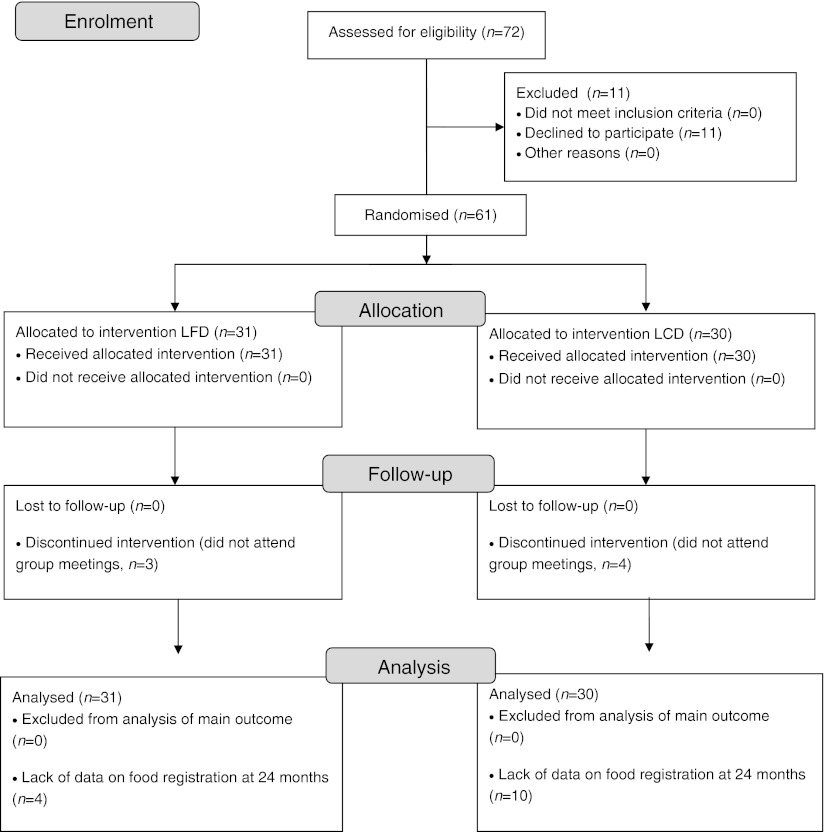
Flow diagram of the study

The study nurses invited 72 consecutive patients to participate in the study from the autumn of 2008 to the start of the clinical trial in March 2009. Ten patients declined because they judged the study to be too time-consuming and one patient believed that a high-fat diet might be hazardous. The remaining 61 patients entered the study, but three in the LFD group and four in the LCD group expressed that they had severe difficulty following the prescribed diets and were not willing to participate in the group meetings. Data on the main outcomes from these seven patients were used in statistical analyses according to intention-to-treat analysis (Table 1; see also Fig. 1 for flow diagram). In the low-fat group, the mean age was 62.7 ± 11 years, there were 13 men and 18 women and the mean duration of known diabetes was 8.8 ± 6.2 years. Corresponding figures for the low-carbohydrate group were 61.2 ± 9.5 years, 14 men and 16 women and a duration of known diabetes of 9.8 ± 5.5 years. Age, sex distribution and known duration of diabetes did not differ between the groups (all *p* > 0.5). At baseline, two patients in the low-fat group and two in the low-carbohydrate group were treated with diet only, 13 in the low-fat group and 15 in the low-carbohydrate group were using oral glucose-lowering medication only, and 11 in the low-fat group and 10 in the low-carbohydrate group were treated with a combination of insulin and oral medication. At 24 months, 14 individuals (four in the LFD group and ten in the LCD group) did not provide diet records, but data on the main outcomes were used in calculations. The dietitian had individual consultations with four patients in the low-carbohydrate group and three patients in the low-fat group; information was otherwise only provided during the group meetings. These individual contacts all took part during the first 12 months of the study. Table 1 shows anthropometrics, laboratory variables and medication from baseline throughout the study. The recorded intake of energy from fat and carbohydrate differed between the groups (Table 2).

**Table 1:** Anthropometrics, metabolic outcomes and medication at 0, 6, 12 and 24 months after the initiation in patients with type 2 diabetes randomised to the LFD (*n* = 31) or LCD (*n* = 30)

Variable	Diet	Time point (months)								*p* value^c^	*p* value^d^
		0		6		12		24			
		Value	*p* value^a^	Value	*p* value^b^	Value	*p* value^b^	Value	*p* value^b^		
Weight (kg)	LFD	98.8 ± 21	0.15	94.2 ± 21	<0.001	94.9 ± 21	<0.001	95.9 ± 21	0.002	<0.001	0.33
	LCD	91.4 ± 19		87.5 ± 19	<0.001	89.5 ± 19	<0.001	89.4 ± 22	0.020	<0.001	
BMI (kg/m^2^)	LFD	33.8 ± 5.7	0.11	32.3 ± 5.5	<0.001	32.6 ± 5.3	<0.001	32.8 ± 5.5	0.002	<0.001	0.20
	LCD	31.6 ± 5.0		30.1 ± 5.1	<0.001	30.7 ± 5.3	<0.001	30.8 ± 5.8	0.011	<0.001	
Waist (cm)	LFD	110 ± 13	0.29	106 ± 15	<0.001	106 ± 14	<0.001	108 ± 16	0.035	<0.001	0.42
	LCD	106 ± 15		102 ± 14	<0.001	104 ± 15	0.021	104 ± 16	0.015	0.002	
Sagittal abdominal diameter (cm)	LFD	27 ± 5	0.37	27 ± 4	0.097	27 ± 4	0.13	28 ± 4	0.62	0.088	0.068
	LCD	26 ± 4		25 ± 4	0.006	25 ± 4	0.006	25 ± 4	0.014	0.002	
HbA_1c_ (%)	LFD	7.2 ± 2.9	0.23	7.2 ± 3.0	0.56	7.3 ± 3.2	0.66	7.4 ± 3.1	0.29	0.40	0.76
	LCD	7.5 ± 3.1		7.1 ± 3.1	0.004	7.3 ± 3.3	0.12	7.5 ± 3.1	0.98	0.005	
HbA_1c_ (mmol/mol)	LFD	55.6 ± 8.0	0.23	54.7 ± 9.7	0.56	56.4 ± 11.4	0.66	57.6 ± 10.8	0.29	0.40	0.76
	LCD	58.5 ± 10.2		53.7 ± 10.3	0.004	56.2 ± 12.4	0.12	58.4 ± 10.6	0.98	0.005	
Systolic blood pressure (mmHg)	LFD	136 ± 13	0.73	128 ± 12	<0.001	126 ± 12	<0.001	125 ± 13	<0.001	<0.001	0.74
	LCD	135 ± 15		126 ± 17	0.004	127 ± 13	0.003	126 ± 14	0.012	0.003	
Diastolic blood pressure (mmHg)	LFD	77 ± 9	0.67	74 ± 8	0.049	69 ± 9	<0.001	71 ± 11	0.001	<0.001	0.75
	LCD	76 ± 11		72 ± 8	0.019	70 ± 10	0.002	71 ± 8	0.004	0.002	
Total cholesterol (mmol/l)	LFD	4.3 ± 1.0	0.40	4.2 ± 1.1	0.91	4.3 ± 1.1	0.96	4.0 ± 0.9	0.11	0.23	0.33
	LCD	4.5 ± 1.0		4.4 ± 1.1	0.60	4.3 ± 0.9	0.17	4.4 ± 0.9	0.32	0.63	
LDL-cholesterol (mmol/l)	LFD	2.4 ± 0.7	0.24	2.3 ± 0.8	0.69	2.3 ± 0.8	0.48	2.1 ± 0.7	0.017	0.050	0.16
	LCD	2.7 ± 0.9		2.5 ± 0.7	0.37	2.5 ± 0.8	0.12	2.4 ± 0.7	0.020	0.13	
HDL-cholesterol (mmol/l)	LFD	1.09 ± 0.29	0.57	1.10 ± 0.30	0.36	1.17 ± 0.24	0.004	1.20 ± 0.32	0.002	0.001	0.15
	LCD	1.13 ± 0.33		1.25 ± 0.47	0.018	1.24 ± 0.38	0.024	1.36 ± 0.44	<0.001	<0.001	
Triacylglycerols (mmol/l)	LFD	1.8 ± 0.8	0.89	1.8 ± 1.3	0.79	1.7 ± 0.9	0.88	1.7 ± 0.9	0.81	0.98	0.35
	LCD	1.7 ± 1.4		1.5 ± 1.2	0.39	1.4 ± 0.8	0.20	1.5 ± 0.8	0.22	0.68	
Total insulin dose (E)	LFD	39 ± 51	0.86	38 ± 48	0.12	38 ± 48	0.29	36 ± 44	0.50	0.81	0.83
	LCD	42 ± 65		30 ± 47^e^	0.020	33 ± 54	0.041	35 ± 56	0.14	0.007	
Metformin (mg)	LFD	1,435 ± 946	0.80	1,274 ± 884	0.096	1,371 ± 875	0.40	1,306 ± 901	0.28	0.23	0.93
	LCD	1,375 ± 950		1,442 ± 872^e^	0.29	1,358 ± 915	0.86	1,292 ± 911	0.38	0.39	
Glibenclamide (mg)	LFD	0.4 ± 1.9	0.26	0.3 ± 1.3	0.33	0.3 ± 1.3	0.66	0.3 ± 1.3	0.66	0.69	0.56
	LCD	1.1 ± 2.6		0.5 ± 1.3	0.057	0.5 ± 2.0	0.24	0.1 ± 0.7	0.055	0.082	
Simvastatin (mg)	LFD	19 ± 17	1.00	19 ± 17	-^f^	24 ± 17	0.032	24 ± 17	0.032	0.003	0.54
	LCD	19 ± 18		23 ± 19	0.096	28 ± 20	0.004	27 ± 21	0.008	0.001	
Atorvastatin (mg)	LFD	2 ± 5	0.97	2 ± 6	0.33	3 ± 9	0.18	3 ± 9	0.18	0.24	0.88
	LCD	2 ± 5		2 ± 6	0.33	2 ± 6	0.33	2 ± 6	0.33	0.40	

^a^Between groups at baseline

^b^For change compared with baseline

^c^For change over all time points

^d^For change over all time points between groups

^e^Denotes statistically significant difference of change compared with baseline between the groups

^f^As there were no changes in simvastatin dose, the *t* test is not applicable

**Table 2:** Dietary outcomes at 0, 6, 12 and 24 months after the initiation in patients with type 2 diabetes randomised to the LFD or LCD

Variable	0 months	3–6 months		12 months		24 months		*p* value^b^	*p* value^c^
	*n* = 61	*n* = 55		*n* = 42		*n* = 47			
	Value	Value	*p* value^a^	Value	*p* value^a^	Value	*p* value^a^		
Energy intake (kJ)									
LFD	7,569 ± 2,063	6,498 ± 1,787	0.005	6,619 ± 2,075	0.075	6,104 ± 1,891	0.002	0.010	0.065
LCD	7,071 ± 1,782	5,791 ± 1,531	<0.001	6,017 ± 2,075	0.037	5,234 ± 1,799	<0.001	<0.001	
Carbohydrate energy (%)									
LFD	48 ± 6	49 ± 6	0.88	47 ± 6	0.38	47 ± 7	0.31	0.28	<0.001
LCD	41 ± 11^e^	25 ± 8^d^	<0.001	27 ± 8^d^	<0.001	31 ± 6^d^	<0.001	<0.001	
Fat energy (%)									
LFD	32 ± 5	29 ± 5	0.12	31 ± 6	0.96	31 ± 7	0.87	0.28	<0.001
LCD	39 ± 7^e^	49 ± 7^d^	<0.001	47 ± 6^d^	<0.001	44 ± 5^d^	<0.001	<0.001	
Protein energy (%)									
LFD	19 ± 3	21 ± 3	0.012	20 ± 3	0.044	20 ± 2	0.045	0.037	0.009
LCD	19 ± 3	24 ± 3^d^	<0.001	23 ± 5^d^	0.002	24 ± 4^d^	<0.001	<0.001	
Alcohol energy (%)									
LFD	1 ± 2	2 ± 3	0.23	2 ± 3	0.36	2 ± 3	0.27	0.72	0.49
LCD	2 ± 4	2 ± 4	0.83	2 ± 3	0.037	2 ± 4	0.79	0.018	
Total fat (g)									
LFD	66 ± 23	53 ± 20	0.014	56 ± 23	0.27	52 ± 22	0.007	0.11	0.008
LCD	74 ± 23	78 ± 24^d^	0.081	77 ± 29	0.22	63 ± 24	0.17	0.10	
Saturated fat energy (%)									
LFD	13 ± 3	11 ± 2	0.090	12 ± 3	0.96	13 ± 3	0.61	0.20	<0.001
LCD	16 ± 4	20 ± 4^d^	<0.001	20 ± 4^d^	0.008	19 ± 2^d^	<0.001	<0.001	
Unsaturated fat energy (%)									
LFD	12 ± 2	11 ± 2	0.20	11 ± 2	0.71	11 ± 3	0.50	0.80	<0.001
LCD	14 ± 3	18 ± 3^d^	<0.001	17 ± 3^d^	<0.001	16 ± 3^d^	<0.001	<0.001	
Polyunsaturated fat energy (%)									
LFD	5 ± 2	5 ± 2	0.92	5 ± 2	0.97	5 ± 2	0.58	0.76	0.001
LCD	6 ± 3	8 ± 2^d^	<0.001	8 ± 2^d^	0.006	6 ± 2	0.044	0.002	

Data are given for all participants who provided complete diet records

^a^Compared with baseline

^b^For change over all time points

^c^For change over all time points (between groups)

^d^Denotes statistically significant difference between changes in the two groups

^e^Denotes statistically significant difference between groups at baseline

**Fig. 2:**
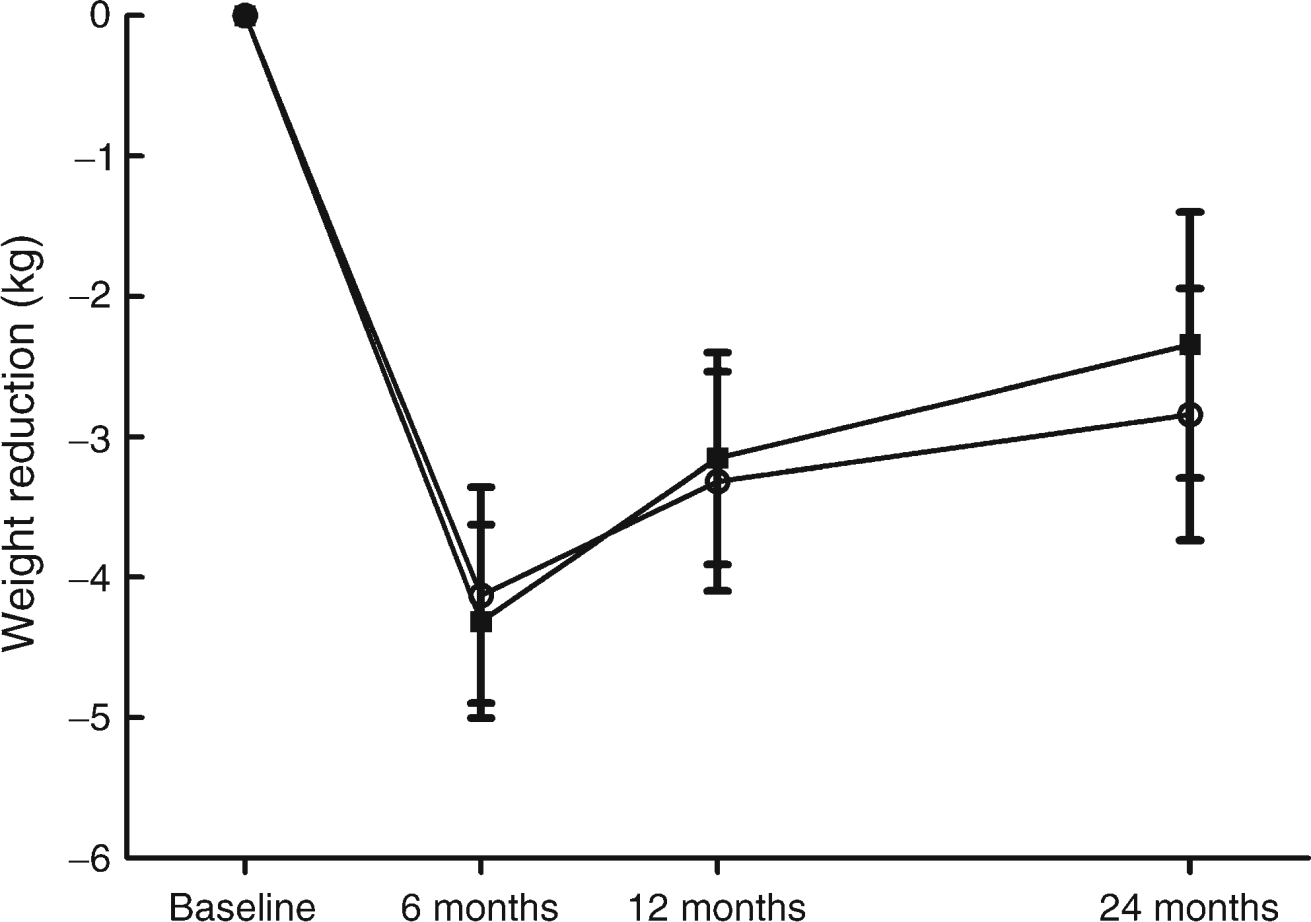
Comparison of weight reduction with an LCD, aiming to achieve 20 E% from carbohydrate (squares), and an LFD (circles), aiming for 55–60 E% from carbohydrates, during 2 years in patients with type 2 diabetes. The weight reduction did not differ between the groups (*p* = 0.33 for all time points)

There was no difference in weight reduction between the groups at 6 months (LFD group −4.0 ± 4.1 kg; LCD group −4.3 ± 3.6 kg; *p* = 0.75 for difference in change between groups and *p* < 0.001 within either group; Table 1 and Fig. 2). There were also no statistically significant differences in weight reduction between groups after adjustment for baseline carbohydrate or fat intake (all *p* > 0.5).

**Fig. 3:**
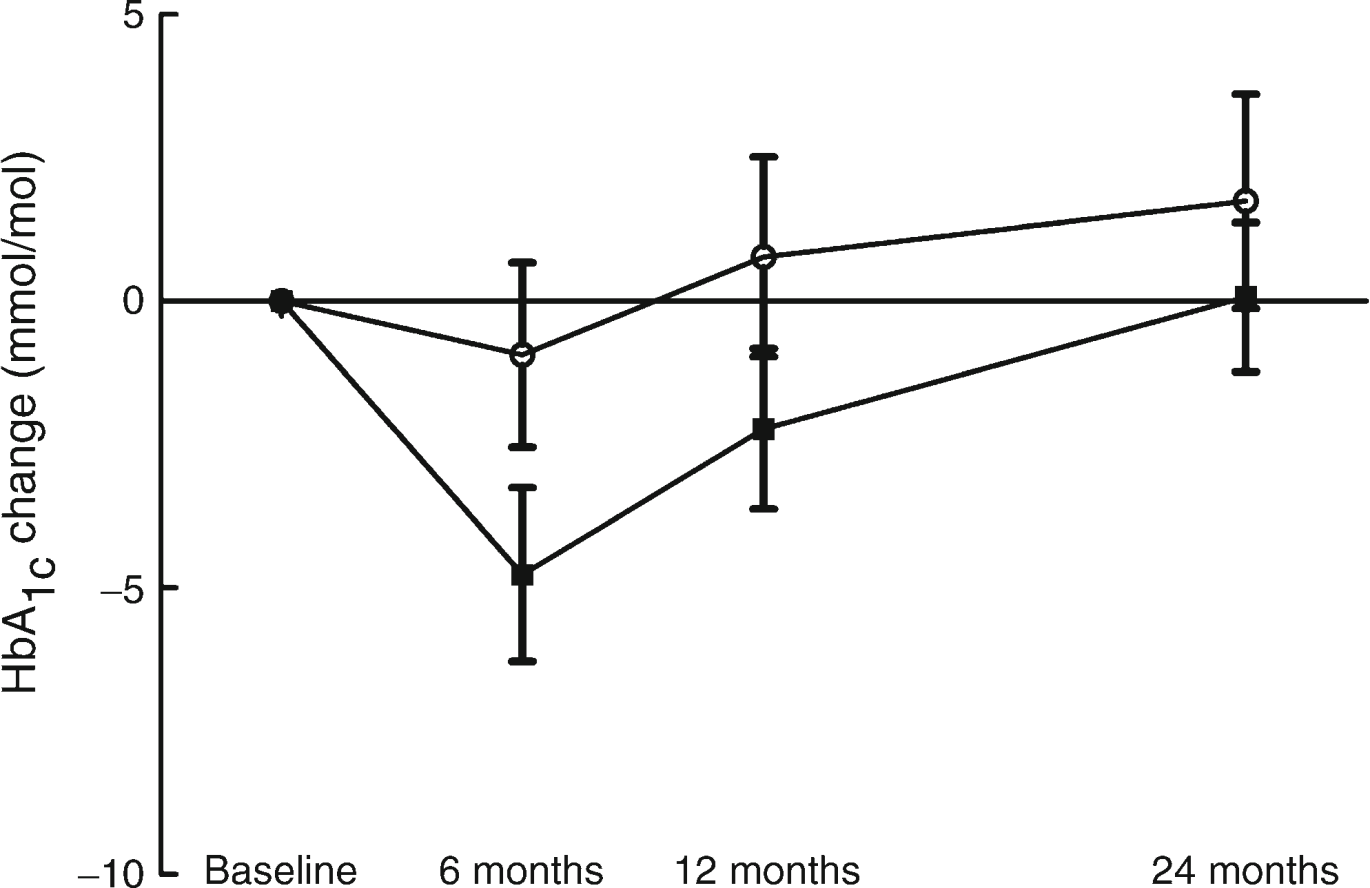
Comparison of the reduction in HbA_1c_ levels following an LCD with the aim of achieving 20 E% from carbohydrate (squares), and an LFD (circles), aiming for 55–60 E% from carbohydrate, over 2 years in patients with type 2 diabetes. The reduction in HbA_1c_ level was statistically significant within the low-carbohydrate group (*p* = 0.005 for all time points), but did not differ between the groups when compared at all time points (*p* = 0.76). To convert values for HbA_1c_ in mmol/mol into %, divide by 10.929 and add 2.15

A significant reduction in HbA_1c_ was seen at 6 months in the low-carbohydrate group only (Table 1). However, HbA_1c_ levels gradually returned to baseline levels after 6 months as shown in Fig. 3 and Table 1. The change in HbA_1c_ level at 6 months compared with baseline was not statistically significant between the groups (*p* = 0.089). Reductions in oral medication and insulin dose were made consecutively to avoid hypoglycaemia, and the reduction in insulin was statistically significant only in the low-carbohydrate group at 6 months (Table 1). This change in the average insulin dose was statistically significant between the two groups at 6 months (*p* = 0.046).

There were no significant differences between groups regarding office blood pressure levels in the study (Table 1). At 6 months, four patients in the LCD group and one patient in the LFD group had started on treatment with statins or had their former dose increased (see also Table 1). Corresponding figures for these medical adjustments during the entire study period were nine patients in the LCD group and seven patients receiving the LFD. By the end of the trial, 54 of 61 patients received lipid-lowering medication. At 6 months, the low-carbohydrate group showed significantly increased levels of HDL-cholesterol (Table 1; *p* = 0.018 for change within group, *p* = 0.077 for change between groups). No patient suffered cardiovascular disease or other serious adverse events during the study.

The results of the dietary records are shown in Table 2. During the first 6 months, adherence to the proposed diet was comparatively good in both groups as judged by mean values of macronutrient intake (Table 2). The low-fat group did not significantly change the macronutrient composition during the study, while there was an increase in energy from fat in those randomised to LCD (Table 2). Despite reduction of energy intake, total intake of fat showed no reduction in the low-carbohydrate group, which was in contrast to the low-fat group (Table 2). The percentage of energy intake from saturated fat increased in the low-carbohydrate group throughout the whole study, but there were no differences between the groups regarding energy intake, according to the diet records (Table 2).

### Completers analysis

Additional analyses were made according to compliance with energy intake. Table 3 shows results for only those patients who consumed ≤6,694 kJ/day (1,600 kcal/day) for women or ≤7,531 kJ/day (1,800 kcal/day) for men according to the last diet record at 24 months; it includes 17 patients in the low-fat group and 18 patients in the low-carbohydrate group. At 24 months the patients randomised to the LFD weighed 3.1 ± 4.3 kg less than at baseline and the corresponding figure for the low-carbohydrate group was 3.5 ± 4.0 kg. This suggests that, in particular, the high-fat group had lower compliance at this time point, which affected weight gain in the total LCD-cohort (i.e. −3.5 ± 4.0 kg in completers compared with −2.3 ± 5.1 kg in the total cohort). While sagittal abdominal diameter was stable in the low-fat group, this was reduced by 2 cm in the low-carbohydrate group. However, the low-fat group with energy compliance had reduced systolic and diastolic blood pressure, effects lacking in those randomised to the high-fat diet (Table 3). A second completers analysis based on compliance with fat intake is available in the electronic supplementary material (ESM; compliance defined as: ≤35 E% from fat in the low-fat group, *n* = 20, or ≥45 E% from fat for the low-carbohydrate group, *n* = 12, *χ* ^2^ between groups for compliance with intake of fat *p* = 0.06). Similar results to those in the completers analysis according to energy intake were found in this post-hoc calculation for sagittal abdominal diameter and blood pressure. HDL-cholesterol increased from 1.11 ± 0.36 to 1.46 ± 0.59 mmol/l (*p* = 0.001 for all time points) in those compliant with the LCD according to fat intake, while corresponding figures for the LFD were 1.05 ± 0.26 mmol/l at baseline to 1.17 ± 0.25 mmol/l at 24 months (*p* = 0.006 within group and *p* = 0.016 for change between groups).

**Table 3:** Completers analysis of anthropometrics, metabolic outcomes and medication in patients with type 2 diabetes randomised to the LFD or LCD who were compliant with energy restriction <6,694 kJ/day (1,600 kcal/day) for women or <7,531 kJ/day (1,800 kcal/day) for men according to diet records at the 24 month registration

Variable	Diet	Time point (months)								*p* value^c^	*p* value^d^
		0		6		12		24			
		Value	*p* value^a^	Value	*p* value^b^	Value	*p* value^b^	Value	*p* value^b^		
Weight (kg)	LFD	90.6 ± 19	0.66	86.7 ± 19	<0.001	88.0 ± 19	<0.001	87.5 ± 19	0.008	<0.001	0.75
	LCD	88.0 ± 16		83.4 ± 15	<0.001	85.6 ± 15	<0.001	84.4 ± 16	0.002	<0.001	
BMI (kg/m^2^)	LFD	31.6 ± 5	0.71	30.2 ± 5	<0.001	30.7 ± 5	<0.001	30.5 ± 5	0.005	<0.001	0.74
	LCD	31.0 ± 4.5		29.4 ± 4.1	<0.001	30.0 ± 4.5	0.001	29.8 ± 4.5	0.002	<0.001	
Waist (cm)	LFD	107 ± 13	0.31	102 ± 15	<0.001	103 ± 16	<0.001	103 ± 16	0.004	<0.001	0.57
	LCD	103 ± 12		100 ± 10	0.004	100 ± 10	0.072	100 ± 12	0.003	0.023	
Sagittal abdominal diameter (cm)	LFD	26 ± 5	0.97	25 ± 3	0.58	26 ± 3	0.68	26 ± 4	0.49	0.46	0.40
	LCD	26 ± 4		25 ± 3	0.009	24 ± 3	0.003	24 ± 3	0.016	0.002	
HbA_1c_ (%)	LFD	7.4 ± 2.8	0.83	7.3 ± 3.1	0.23	7.2 ± 2.9	0.021	7.5 ± 3.1	0.69	0.19	0.73
	LCD	7.5 ± 2.8		7.0 ± 2.9	0.016	7.2 ± 3.1	0.062	7.5 ± 2.9	0.94	0.026	
HbA_1c_ (mmol/mol)	LFD	57.9 ± 7.6	0.83	55.9 ± 9.9	0.23	55.5 ± 8.0	0.021	58.5 ± 10.4	0.69	0.19	0.73
	LCD	58.4 ± 7.5		52.8 ± 8.3	0.016	54.8 ± 10.1	0.062	58.3 ± 8.7	0.94	0.026	
Systolic blood pressure (mmHg)	LFD	134 ± 11	0.73	129 ± 13	0.078	127 ± 12	0.022	123 ± 10	0.005	0.007	0.73
	LCD	133 ± 13		125 ± 16	0.053	127 ± 13	0.085	126 ± 14	0.195	0.13	
Diastolic blood pressure (mmHg)	LFD	74 ± 10	0.91	74 ± 8	0.84	68 ± 9	0.035	70 ± 8	0.032	0.012	0.80
	LCD	74 ± 11		71 ± 8	0.17	71 ± 11	0.16	71 ± 8	0.11	0.22	
Total cholesterol (mmol/l)	LFD	4.0 ± 0.7	0.078	4.1 ± 0.9	0.67	4.0 ± 0.7	0.66	3.9 ± 0.8	0.57	0.73	0.11
	LCD	4.5 ± 1.0		4.4 ± 1.3	0.65	4.4 ± 0.9	0.34	4.4 ± 1.0	0.67	0.90	
LDL-cholesterol (mmol/l)	LFD	2.2 ± 0.4	0.043	2.2 ± 0.7	0.84	2.2 ± 0.6	0.91	2.0 ± 0.7	0.16	0.43	0.13
	LCD	2.7 ± 0.9		2.5 ± 0.9	0.37	2.5 ± 0.7	0.10	2.4 ± 0.8	0.066	0.34	
HDL-cholesterol (mmol/l)	LFD	1.14 ± 0.32	0.94	1.18 ± 0.32	0.28	1.21 ± 0.26	0.029	1.26 ± 0.34	0.050	0.080	0.67
	LCD	1.15 ± 0.36		1.26 ± 0.48	0.034	1.23 ± 0.38	0.017	1.37 ± 0.46	<0.001	<0.001	
Triacylglycerols (mmol/l)	LFD	1.5 ± 0.7	0.88	1.4 ± 0.7	0.67	1.4 ± 0.7	0.17	1.6 ± 1.0	0.52	0.49	0.91
	LCD	1.4 ± 0.6		1.4 ± 1.1	0.97	1.4 ± 0.5	0.84	1.5 ± 0.8	0.56	0.96	
Total insulin dose (E)	LFD	32 ± 41	0.73	30 ± 37	0.34	31 ± 38	0.73	30 ± 40	0.68	0.92	0.38
	LCD	26 ± 54		14 ± 28	0.13	16 ± 33	0.16	20 ± 37	0.34	0.12	
Metformin (mg)	LFD	1,353 ± 981	0.81	1,176 ± 865	0.27	1,324 ± 847	0.79	1,265 ± 903	0.65	0.58	0.90
	LCD	1,278 ± 844		1,444 ± 784	0.055	1,306 ± 860	0.85	1,222 ± 826	0.71	0.37	
Glibenclamide (mg)	LFD	0.7 ± 2.6	0.45	0.5 ± 1.7	0.33	0.6 ± 1.7	0.67	0.6 ± 1.7	0.67	0.69	1.0
	LCD	1.5 ± 3.1		0.5 ± 1.2	0.056	0.3 ± 0.9	0.083	0.2 ± 0.8	0.099	0.039	
Simvastatin (mg)	LFD	21 ± 17	0.64	21 ± 17	–^e^	25 ± 17	0.19	25 ± 17	0.19	0.14	0.95
	LCD	18 ± 19		23 ± 19	0.16	26 ± 23	0.049	26 ± 23	0.049	0.025	
Atorvastatin (mg)	LFD	1 ± 2	0.21	1 ± 5	0.33	1 ± 5	0.33	1 ± 5	0.33	0.40	0.30
	LCD	3 ± 7		3 ± 8	0.33	3 ± 8	0.33	3 ± 8	0.33	0.40	

LFD, *n* = 17; LCD, *n* = 18

^a^Between groups at baseline

^b^For change compared with baseline

^c^For change over all time points

^d^For change over all time points between groups

^e^As there were no changes in simvastatin doses, the *t* test is not applicable

## Discussion

Our study did not confirm the finding that weight reduction is more efficient in individuals following an LCD than in those following an LFD, as has been found in some previous trials [6, 9, 12, 18–20]. An important difference in our study compared with these earlier studies [6, 9, 12, 18–20] was that we used comparatively fewer resources to achieve compliance. In our study, only four group meetings were offered during the first 12 months of the trial. The rationale for this design was to make the results more applicable to regular clinic care in which educational activities such as group meetings can be offered as a means to improve glycaemic control. No patients were lost to follow-up and data on glycaemic control were complete at 24 months, while data on weight were lacking for only one participant at this time point. This outcome of our study left minimal room for the selection of participants, who did indeed find either of the diets suitable, to affect the main outcomes. Our findings indicate that if patients are randomised to an LCD compared with an LFD with resources used to achieve changes in diet composition that are readily available for many providers of care, both diets induce similar weight reductions. This was also in line with the finding that both groups reported similar energy intake during the study. Westman et al have reported more efficient weight reduction with an LCD after 6 months when compared with an LFD [9]. In that study, diet information was facilitated, compared with our design, by lack of energy restriction in the low-carbohydrate group. This could have affected the more beneficial findings compared with our study regarding weight loss. Also, Westman et al had a total of 18 group meetings during their 6 month study, and this could have affected the outcome. Information on increased exercise was also part of their lifestyle change programme, but was not included in ours. Specifically, we aimed to study the effects of macronutrient composition on glycaemic control and cardiovascular risk factors, which was why we aimed to achieve no differences in energy intake in the information we gave to the participants. Interestingly, we did find an increase in HDL-cholesterol after 6 months and a specific reduction in HbA_1c_ levels in the low-carbohydrate group only, which suggests that these effects are dependent on macronutrient composition per se; this is in line with the findings of Westman et al [9].

We also acknowledge that we might have achieved better weight reduction if a design similar to that used in ‘Weight Watchers’ programmes had been incorporated. For regular care provided by the Swedish tax-based system, incentives used in Weight Watchers, such as public display of the body weight results of the participants, would not be feasible for general use in clinic care because of patient privacy. Also, one should keep in mind that there is selection and incentive in such commercially run programmes, as participants are willing to pay to participate. However, effective weight loss in a Weight Watchers group was recently shown in a study even when the cost for participation was reimbursed by the study organisers [21].

Although patients in our study who had been randomised to the low-carbohydrate group reported a lower intake of carbohydrates at baseline compared with the low-fat group, this was unrelated to weight changes in statistical analyses. In retrospect, this group difference in reported intake of macronutrients between the groups might have been a consequence of the participants being informed of the randomisation results before the diet record at baseline was performed. Consequently, some participants may have adjusted their diet to make it similar to that to which they had been allocated, ahead of the first group information meeting. Unfortunately the baseline difference was not elucidated until the end of the trial and it was thus judged to be of little meaning to ask participants with little intake of energy from carbohydrates in the low-carbohydrate group at baseline whether this was a consequence of the randomisation, which had occurred more than 2 years earlier.

The largest changes in macronutrient intake were seen in patients randomised to the low-carbohydrate group. Indeed, patients in the low-fat group had the same macronutrient composition at baseline as during the study, suggesting that this was indeed a traditional diet and that they, according to the diet records, had been given similar diet recommendations earlier.

The patients following the LCD increased the percentage of energy intake from both total and saturated fat throughout the 24 months of the trial according to diet records, in line with the study protocol. At 6 months, when weight reduction was most pronounced, only the LCD group had changes in blood lipid levels in the form of increased HDL-cholesterol. However, during the study there had also been changes in lipid-lowering therapy that make these findings inconclusive regarding whether they solely depended on changes in diet. At the end of the trial, several patients had been newly started on lipid-lowering therapy. This is an obvious limitation of our trial from a mechanistic point of view, but it was a consequence of our efforts to limit the resources necessary for the diets to be implemented in regular primary care, to allow the methods to translate easily to real-life application. However, as 3-hydroxy-3-methylglutaryl-coenzyme A reductase inhibitors (statins) mainly affect LDL-cholesterol levels and, as earlier trials have also found that diets high in fat elevate HDL-cholesterol to a greater extent than high-carbohydrate diets in type 2 diabetes [9, 11, 16], we find it likely that the increase in HDL-cholesterol in our trial was mainly an effect of the change in diet.

We acknowledge that the general applicability of our study results might be limited because of the high participation rate that was achieved. The study nurses had also taken care of the same patients ahead of the study start and when identifying potential participants according to inclusion and exclusion criteria it cannot be excluded that, prior to the study, they might have discharged patients judged not to have been suitable participants for various reasons. Another potential explanation for the high participation rate was that the study protocol was not very time-consuming for the patients as it involved only four group meetings. We also acknowledge the problems with diet records. Although diet records with notebooks and scales can be more detailed and precise than standardised food frequency questionnaires, results from surveys of food intake have low reproducibility and, in particular, there are systematic errors in underreporting energy intake [22]. Thus, total energy intake might not be accurate in our study, but the lowering of HbA_1c_ in only the low-carbohydrate group at 6 months and also differences in HDL-cholesterol changes at similar weight reductions suggest that the groups did indeed change their macronutrient intake differently in our trial.

The analyses of outcome in the participants who were compliant with either energy intake or with the E% from fat implied better long-term effects on weight loss than in the total cohort analysed on an intention-to-treat basis. Although this was a post-hoc analysis, and thus data should be interpreted with caution, it was of interest to note that HDL-cholesterol increased by 33% in patients reasonably compliant with fat intake, which was in line with data from Westman et al [9]. However, blood pressure levels were not reduced in patients on the LCD at 24 months. It cannot be excluded that salt intake increased in parallel with ingestion of fat, as has been demonstrated in the general population [23], leading to less favourable blood pressure levels. Unfortunately, we did not collect urine for determination of the amount of sodium.

In conclusion, our findings support the use of an LCD with 20 E% from carbohydrates as an alternative to a traditional low-fat diet, if the aim primarily is to improve glycaemic control in type 2 diabetes. We achieved a weight loss of about 4 kg in both groups after 6 months based on group information on three occasions and there was only one more group meeting, which took place at 12 months’ study duration. However, as in many earlier studies, compliance with the LCD was reduced after 6 months, as judged by the increase in body weight and according to food records, and it cannot be ruled out that different results could have been obtained if more effort had been made to achieve compliance with the diet composition and reduction of energy intake.

## Electronic supplementary material

Below is the link to the electronic supplementary material.

ESM Table 1 PDF 98 kb

## Abbreviations

E%: Energy per cent
LCD: Low-carbohydrate diet
LFD: Low-fat diet
