# Supplementary material for: In type 2 diabetes, randomisation to advice to follow a low-carbohydrate diet transiently improves glycaemic control compared with advice to follow a low-fat diet producing a similar weight loss
Source: Diabetologia. 2012 May 6;55(8):2118–27. doi: 10.1007/s00125-012-2567-4 (PMC3390696; doi:10.1007/s00125-012-2567-4)
Supplement: Supplementary file 1 — PDF 98 kb [file 125_2012_2567_MOESM1_ESM.pdf]

Electronic supplementary material

Table 1

Anthropometrics, metabolic outcomes and medication at 0, 6, 12 and 24 months after the initiation in patients with type 2 diabetes randomised to a low-fat or low-carbohydrate diet and had followed the fat-restriction: ≤35 energy % for low-fat group (n= 20), ≥45 energy % for low-carbohydrate group (n= 12) at the 24 month registration.

**Variable**	**Diet**	**Time point**									
		**0 months**		**6 months**		**12 months**		**24 months**			
			P value[a]		*P* value[b]		*P* value[b]		*P* value[b]	*P* value[c]	*P* value[d]
Weight (kg)	Low-fat	98.4±19	0.061	93.8±19	<0.001	94.5±18	0.002	94.9±19	0.001	<0.001	0.096
	Low-carb.	85.0±18		80.1±16	<0.001	82.8±17	0.001	82.0±18	0.018	<0.001	
BMI (kg/m^2)	Low-fat	34.8±5.6	0.040	33.1±5.2	<0.001	33.4±5.0	0.002	33.5±5.6	0.001	<0.001	0.053
	Low-carb.	30.4±5.4		28.7±4.9	<0.001	29.3±5.4	0.001	29.3±5.4	0.016	<0.001	
Waist (cm)	Low-fat	111±12	0.033	107±13	0.001	107±12	<0.001	108±14	0.011	<0.001	0.072
	Low-carb.	102±13		98±11	0.002	97±11	0.003	100±13	0.022	<0.001	
Sagittal abdominal diameter (cm)	Low-fat	27±5	0.11	27±4	0.24	27±4	0.27	27±4	0.98	0.40	0.010
	Low-carb.	25±4		23±3	0.008	23±3	0.006	23±4	0.013	0.003	
HbA1c (%) (mmol/mol)	Low-fat	7.1±2.9<br>54.5±8.0	0.28	7.0±3.0<br>52.9±9.7	0.35	6.9±3.0<br>52.3±9.1	0.16	7.4±3.2<br>56.9±11.1	0.22	0.021	0.77
	Low-carb.	7.4±2.7<br>57.5±6.4		6.9±3.1<br>51.6±10.0	0.034	7.1±3.2<br>54.5±11.3	0.16	7.3±2.8<br>56.5±7.4	0.65	0.14	
Systolic blood pressure (mmHg)	Low-fat	137±13	0.065	127±14	0.006	125±10	0.001	125±15	0.001	0.001	0.11
	Low-carb.	128±14		122±16	0.22	125±12 [f]	0.29	122±13	0.44	0.51	
Diastolic blood pressure (mmHg)	Low-fat	78±8	0.054	75±7	0.065	67±9	<0.001	72±11	0.014	<0.001	0.34
	Low-carb.	71±13		70±9	0.72	70±11 [f]	0.57	68±6	0.20	0.39	

Total cholesterol (mmol/l)	Low-fat	4.2±0.9	0.59	4.2±1.0	0.78	4.3±1.1	0.31	4.0±0.9	0.44	0.46	0.86
	Low-carb.	4.0±0.9		4.1±1.2	0.59	4.2±0.8	0.38	4.2±1.0	0.38	0.79	
LDL-cholesterol (mmol/l)	Low-fat	2.3±0.6	0.89	2.4±0.8	0.63	2.4±0.8	0.70	2.1±0.7	0.13	0.18	0.89
	Low-carb.	2.3±0.8		2.3±0.9	0.96	2.3±0.6	0.77	2.2±0.7	0.39	0.67	
HDL-cholesterol (mmol/l)	Low-fat	1.05±0.26	0.63	1.09±0.29	0.10	1.16±0.22	0.005	1.17±0.25	0.003	0.006	0.18
	Low-carb.	1.11±0.36		1.33±0.57	0.035	1.29±0.47	0.059	1.46±0.59 [f]	0.007	0.001	
Triglycerides (mmol/l)	Low-fat	1.7±0.8	0.091	1.6±0.7	0.30	1.7±0.9	0.98	1.6±0.7	0.39	0.53	0.060
	Low-carb.	1.3±0.6		1.1±0.4	0.094	1.2±0.4	0.39	1.3±0.8	0.84	0.37	
Total insulin dose (E)	Low-fat	35±56	0.11	32±52	0.17	33±51	0.34	35±50	0.89	0.68	0.10
	Low-carb.	8±17		6±13	0.17	7±14	0.73	10±23	0.63	0.49	
Metformin (mg)	Low-fat	1425±936	0.44	1175±878	0.056	1300±865	0.17	1200±894	0.095	0.063	0.91
	Low-carb.	1167±862		1417±793	0.053	1208±891	0.86	1167±862	1.0	0.83	
Glibenclamide (mg)	Low-fat	0.5±2.3	0.12	0.4±1.6	0.33	0.4±1.6	0.67	0.4±1.6	0.67	0.69	0.43
	Low-carb.	2.2±3.6		0.7±1.4	0.054	0.4±1.1	0.082	0.4±1.0	0.099	0.036	
Simvastatin (mg)	Low-fat	24±18	0.43	24±18	- [e]	26±17	0.33	26±17	0.33	0.40	0.14
	Low-carb.	29±17		33±14	0.34	38±19	0.096	38±19	0.096	0.073	
Atorvastatin (mg)	Low-fat	1±4	0.45	1±4	- [e]	2±9	0.33	2±9	0.33	0.33	0.45
	Low-carb.	0±0		0±0	- [e]	0±0	- [e]	0±0	- [e]	- [e]	

Abbreviations: BMI, body-mass-index; Carb., carbohydrate.

[a]Between groups at baseline
[b]For change compared with baseline
[c]For change over all time points
[d]For change over all time points between groups
[e]As there were no changes in simvastatin doses, the *t* test is not applicable
[f]Statistical significant change between groups at this time point
